# Evidences for Red Pigment Concentrating Hormone (*RPCH*) and Beta-Pigment Dispersing Hormone (*β-PDH*) Inducing Oocyte Meiotic Maturation in the Chinese Mitten Crab, *Eriocheir sinensis*

**DOI:** 10.3389/fendo.2021.802768

**Published:** 2021-12-16

**Authors:** Ling-Ling Wei, Tian-Tian Chen, Bi-Yun Luo, Gao-Feng Qiu

**Affiliations:** National Demonstration Center for Experimental Fisheries Science Education, Key Laboratory of Exploration and Utilization of Aquatic Genetic Resources, Ministry of Education, Key Laboratory of Freshwater Aquatic Genetic Resources, Ministry of Agriculture, Shanghai Engineering Research Center of Aquaculture, Shanghai Ocean University, Shanghai, China

**Keywords:** *Eriocheir sinensis*, *RPCH*, *PDH*, oocyte meiosis, ISH, transcriptome

## Abstract

Red pigment concentrating hormone (RPCH) and pigment dispersing hormone (PDH) are crustacean neuropeptides involved in broad physiological processes including body color changes, circadian rhythm, and ovarian growth. In this study, the full-length cDNA of *RPCH* and *PDH* were identified from the brain of the Chinese mitten crab *Eriocheir sinensis*. The deduced RPCH and PDH mature peptides shared identical sequence to the adipokinetic hormone/RPCH peptides family and the β-PDH isoforms and were designated as Es-RPCH and Es-β-PDH, respectively. *Es-RPCH* and *Es-β-PDH* transcripts were distributed in the brain and eyestalks. The positive signals of *Es-RPCH* and *Es-β-PDH* were localized in the neuronal clusters 6, 8, 9, 10, and 17 of the brain as revealed by *in situ* hybridization. The expression level of *Es-RPCH* and *Es-β-PDH* mRNA in nervous tissues were all significantly increased at vitellogenic stage, and then decreased at the final meiotic maturation stage. The administrated with synthesized Es-RPCH peptide results in germinal vesicles shift toward the plasma membrane in vitellogenic oocyte, and significant decrease of the gonad-somatic index (GSI) and mean oocyte diameter as well as the expression of vitellogenin mRNA at 30 days post injection *in vivo*. Similar results were also found when injection of the Es-β-PDH peptide. *In vitro* culture demonstrated that Es-RPCH and Es-β-PDH induced germinal vesicle breakdown of the late vitellogenic oocytes. Comparative ovarian transcriptome analysis indicated that some reproduction/meiosis-related genes such as cdc2 kinase, cyclin B, 5-HT-R and retinoid-X receptor were significantly upregulated in response to Es-RPCH and Es-β-PDH treatments. Taken together, these results provided the evidence for the inductive effect of *Es-RPCH* and *Es-β-PDH* on the oocyte meiotic maturation in *E. sinensis*.

## Introduction

In most animals, ovarian maturation contains two major cellular events: vitellogenesis and the final meiotic maturation of oocytes, which were precisely regulated by elaborate endocrine system (1–3). Crustacean neuropeptides are important neuroendocrine factors generally synthesized and secreted from the central nerve system (CNS) such as the brain and the X-organ/sinus gland complex in eyestalks, then transported to the target tissue through hemolymph to govern a variety of critical physiological processes including metabolism, molting, growth, and reproduction (4–7). As unique neuroendocrine factors, many crustacean neuropeptides have attracted wide attention in the regulation of ovarian development (1, 4, 8). It has been well known that the neuropeptides in eyestalks exhibit inhibitory effect on the gonadal development. For instances, the crustacean hyperglycemic hormone (CHH) family in eyestalks were well-characterized in the inhibition of *vitellogenin* (*Vg*) expression (9). The neuropeptide F inhibits vitellogenesis and oocyte maturation in the mud crab *Scylla paramamosain* (5). Vitellogenesis-inhibiting hormone (VIH) suppresses vitellogenin production directly in the target tissues and ultimately inhibits the ovarian maturation in the white shrimp *Litopenaeus vannamei* (10). Thus, the removal of eyestalk has become an alternative method for promoting the ovarian maturation in the artificial reproduction of decapod crustaceans (11).

The red pigment concentrating hormone (RPCH) and pigment dispersing hormone (PDH) are another two important crustacean neuropeptides mainly generated in the eyestalks. They have implications in various biological processes including pigment granules concentration or dispersion, body color changes, circadian rhythm and light-dark adaption in crustaceans (12–16). RPCH has been identified as an octapeptide with identical sequence pQLNFSPGWamide in several decapod species such as *Callinectes sapidus* (17), *Scylla olivacea* (18), *Litopenaeus vannamei* (19) and *Penaeus monodon* (20), suggesting it structural conservation among decapod crustaceans. Unlike previously characterized inhibitory hormones in eyestalks, however, functional studies showed that RPCH stimulated the synthesis and release of methyl farnesoate to induce ovarian development in the crayfish *Procambarus clarkii* (21–23). Administrated with RPCH significantly increase the gonad-somatic index and *Vg* expression in the mud crab *S. paramamosain* (24) and the white shrimp *L. vannamei* (19).

In contrast to RPCH, PDH is an octadecapeptide present in multiple forms. Based on the difference of the third amino acid residue at the N-terminus, PDH can be divided into two major categories: α-PDH for the Gly and β-PDH for the Glu (25, 26). To date, the β-PDHs were identified more popular than α-PDH in crustaceans (13, 25, 27–29). Unfortunately, no direct evidence has been provided for the potential role of PDH in ovarian maturation so far, even though the higher expression of *PDH* mRNA level were found in the ovary at vitellogenic stage as compared with other stages in *S. paramamosain* (28). In the present study, we identified the *Es-RPCH* and *Es-β-PDH* transcripts from the brain in the Chinese mitten crab *Eriocheir sinensis*, one of the most important economically aquaculture species in China, and then artificially synthesized Es-RPCH and Es-β-PDH peptides to further functionally characterized their role in regulating ovarian maturation. Our results provide the first evidence for the possible role of *Es-RPCH* and *Es-β-PDH* in the oocyte meiotic maturation thereby providing new potential applications in the mitten crab breeding in aquaculture.

## Materials and Methods

### Animals and Tissue Sampling

Female individuals (body weight 80-110g) were collected from a local farm in Pudong District, Shanghai. The crabs were kept in a freshwater circulation system supplying sufficient dissolved oxygen and were fed on commercial diet pellets (SC-9011, South Ranch, China) once a day. Various tissues including the muscle, gill, heart, hepatopancreas, thoracic ganglia, brain, eyestalks and ovaries were dissected on ice, quickly frozen in liquid nitrogen, and then stored at -80°C for gene expression analysis. For histological observation or *in situ* hybridization analysis, the ovarian and brain tissues were fixed in 4% paraformaldehyde solution at 4°C overnight. The ovarian development was divided into three main stages: previtellogenesis (Pvt), vitellogenesis (Vt) (early, Evt; middle, Mvt; late, Lvt), and the final meiotic maturation (30). Germinal vesicle breaks down (GVBD) in the oocyte at the final meiotic maturation was detected in a clearing solution (31).

### Total RNA Extraction

Total RNA was extracted from tissues using RNAiso Plus reagent (Takara, Kusatsu, Japan) according to the manufacturer’s instructions. The concentration and purity of RNA were quantified by NanoDrop 2000 Spectrophotometers (Thermo Fisher, Massachusetts, USA).

### Rapid Amplification of cDNA Ends (RACE) of *Es-RPCH* and *Es-β-PDH*

The first strand of cDNA was synthesized with 1 μg of total RNA using the SMARTer RACE cDNA Amplification Kit (Clontech, Kusatsu, Japan). Gene-specific primers (**Table S1**) were designed by Primer Premier 5.0 software based on the homologous sequence in the brain transcriptome database (32). RACE-PCR amplification was conducted with initial denaturation at 94°C for 30 s, followed by 35 cycles at 94°C for 5 s, 60°C for 30 s, and 72°C for 2 min, then a final elongation at 72°C for 10 min. PCR products were gel-purified (Tiangen, Beijing, China) and ligated to the pGEM^®^-T Easy vector (Promega, Madison, WI, USA) at 4°C overnight, then transformed into competent *Escherichia coli* (DH5α) cells. Eight positive clones were picked for Sanger sequencing.

### Bioinformatics Analysis

The open reading frame (ORF) of *Es-RPCH* and *Es-β-PDH* were determined using the ORF Finder (https://www.ncbi.nlm.nih.gov/orffinder/). The basic physical and chemical properties of the deduced amino acid sequences were analyzed using the ExPASy Molecular Biology server (https://web.expasy.org/protparam/). The signal peptide was scanned using the SignalP 5.0 Server program (http://www.cbs.dtu.dk/services/SignalP/). Multiple sequence alignment was performed by DNAman software. A phylogenetic tree was generated by MEGAX 64 software using the neighbor-joining methods. Bootstrap analysis of 1000 replicates was carried out to compute the tree branch position confidence (Latin name and protein id are given in **Figure S2**).

### *In Situ* Hybridization (ISH)

DIG-labeled probes of anti-sense and sense RNA were synthesized by DIG RNA Labeling Kit (Roche Diagnostics, Mannheim, Germany) with the SP6, and T7 RNA polymerases (Takara), respectively. ISH was performed as previously described (33). In brief, the brain tissue sections (~6 µm) were dehydrated and deparaffinized with xylene (three times for 5 min each). After rinsing, each section was treated with 3-5 µg/mL proteinase K at 37°C for 10 min, and then hybridized with DIG-labeled antisense or sense RNA probes two hours at 55°C for 2 hours. After serial washing, the DIG was visualized using colorimetric substrates NBT/BCIP (Roche, Germany) following the manufacturer’s instructions.

### *In Vivo* Injection of the Es-RPCH and Es-β-PDH Peptides

The Es-RPCH and Es-β*-*PDH peptides were chemically synthesized (Jier Biosciences, China). Female individuals were randomly divided into four groups: crabs in group 1 were untreated and served as the blank control group, sacrificed after day 0 post injection. Crabs in group 2, 3 and 4 were respectively injected with 100 μL of PBS (control group), Es-RPCH, and Es-β*-*PDH every seven days at the base of the fifth walking leg, and then sacrificed after 30 days post injection. The body weight and gonad weight were measured to calculate the gonadosomatic index (GSI) (gonad weight/body weight × 100%). Ovary tissues from each group were sampled for measuring the relative *Vg* mRNA expression levels and mean oocyte diameter and histological observation.

### *In Vitro* Culture of Oocyte With Es-RPCH and Es-β-PDH Peptides

Ovarian tissues at Lvt stage were selected from the sexually mature crabs (*n = 36*) and then were dispersed with a pipette to release oocytes. The oocytes were rinsed with DPBS and placed in medium 199 in 24-well culture plates as previously described (34) (containing 100 g/mL of BSA, 100 g/mL of streptomycin, and 100 IU/mL of penicillin G). In the experimental group, the synthesized Es-RPCH or Es-β-PDH was added at the different concentrations of 10^-11^ M, 10^-10^ M, 10^-9^ M, 10^-8^ M and 10^-7^ M, respectively, while equal volume of PBS was added to the wells in the control group. All the plates were incubated in dark at 24°C. The number of oocytes in GVBD from each well were determined in clearing solution (formaldehyde, ethyl alcohol, acetic acid, 30:60:1) (31).

### Quantitative Real-Time PCR

Quantitative real-time PCR (qRT-PCR) was performed with gene-specific primers (**Table S1**) using SYBR^®^ Premix Ex Taq™ II kit (Takara) in a 20 µL reaction mix containing 4 µL 5× iScript reaction mix (Bio-Rad), 1 μL cDNA template, 0.5 μL forward primer (10 μM), 0.5 μL reverse primer (10 μM) and 14 μL RNase-free ddH_2_O. Thermal cycling included an initial denaturation at 95°C for 30 s, followed by 40 cycles at 95°C for 5 s and 55°C for 30 s. To examine product specificity, PCR products were verified by sequencing and melting curve analyses (60°C to 95°C in increments of 0.2°C/s), and a negative control was set without cDNA template. Each reaction was conducted in triplicate. The crab *β-actin* gene was employed as an internal reference to estimate the relative mRNA expression levels using the 2^−δδCt^ method (35). The amplification efficiencies for the target gene and the *β-Actin* gene were approximated 100%.

### Statistical Analysis

All data in this study were presented as mean + Standard Deviation (SD), and “*n*” represents the number of replicates. Kolmogorov-Smirnov and Cochran tests were performed and Student’s t-test or Tukey’s test were utilized to analyze the normally distributed data. One-way analysis of variance (ANOVA) and paired sample T-test were performed to calculate statistically significant values respectively using SPSS statistical software (version 20.0). In all cases, statistical differences among groups were accepted at *p <*0.05 and indicated with asterisks or double asterisks.

### RNA-Seq Analysis

The administrated female crabs with PBS, Es-RPCH and Es-β-PDH were sacrificed at 24 hours post injection for sampling ovarian tissues. Three individuals were sampled in each administrated group for RNA-seq libraries construction. RNA-seq were conducted using an Illumina HiSeq™ 2500/MiSeq (Novogene Biotech,Tianjin, China).

Clean reads were obtained after filtering and removing low quality raw reads. The differentially expressed genes (DEGs) between the Es-RPCH and PBS (Es-β-PDH and PBS) groups were screened by DESeq 2 software using DESeq and BH methods with the negative binomial distribution model methods. The screening criteria used for target DEGs is as follows|log2Fold change|≥0, *P* ≤ 0.05. The putative target DEGs were annotated by the Gene Ontology (GO) enrichment analysis (http://www.geneontology.org/), and the pathway was deduced using the Kyoto Encyclopedia of Gene and Genomes (KEGG) database (https://www.kegg.jp/kegg/pathway.html).

## Results

### Molecular Characterization of *Es-RPCH* and *Es-β-PDH*

**Figure 1:**
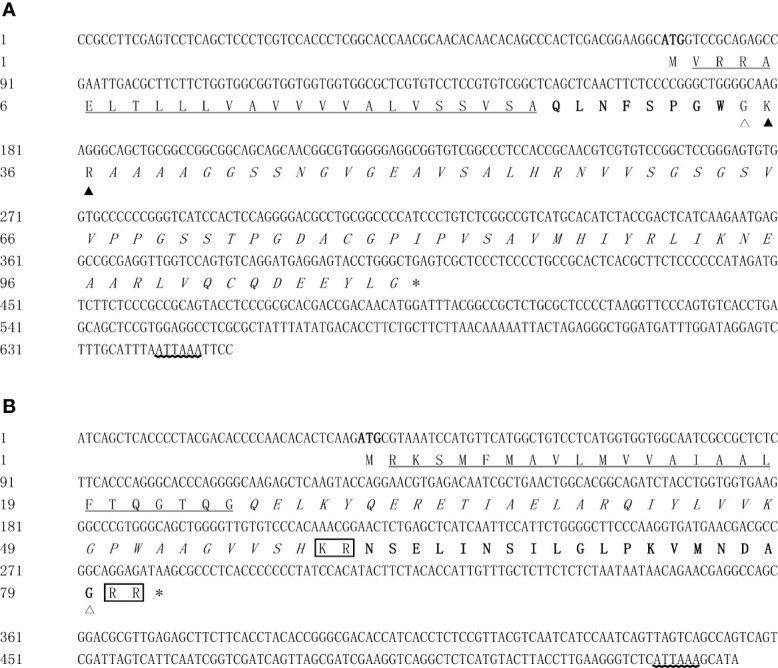
The full-length cDNA and deduced amino acid sequences of *Es-RPCH* **(A)** and *Es-β-PDH* **(B)** in *E. sinensis*. The initiation codon (ATG) and stop codon (TGA/TAA) are indicated in bold and asterisk (*) respectively. The predicted signal peptides are underlined. The putative precursor-related peptides are in italics, and the mature peptides are in bold. The amidation site and potential dibasic cleavage site (KR) of Es-RPCH and two proteolytic sites (KR and RR) of Es-β-PDH are highlighted by white, black triangles and boxes, respectively. The polyadenylation signal (ATTAAA) are indicated by wave lines.

Two full-length cDNA sequences of *Es-RPCH* and *Es-β-PDH* were cloned by RACE method from the brain of *E. sinensis* as shown in **Figure 1**. The 650-base pair (bp) *Es-RPCH* (GenBank accession number: OK315660) contains a 75 bp 5′-untranslated region (UTR), a 245 bp 3′-UTR including a potential polyadenosine (ATTAAA), and a 330 bp ORF encoding a 109 amino acid with a calculated molecular weight of ~11.08 kDa and a predicted isoelectric point of 7.84. Sequence analysis predicted the ORF of Es-RPCH containing a 24-residue signal peptide, a 8-residue mature peptide, and a 73-residue precursor related peptide (RPRP) (**Figure 1A**). Multiple sequence alignments showed that the Es-RPCH amino acid sequence has high identities with other crustacean RPCHs, sharing the identical mature RPCH peptides, glycine residue preceding the dibasic cleavage site, dibasic cleavage site (KR) and the amidation site (**Figure S1A**). Phylogenetic analysis showed that Es-RPCH is more closely related to crustacean RPCHs and clustered with insects AKHs, grouped into one GnRH superfamily, which is consistent with traditional classification (**Figure S2A**).

The *Es-β-PDH* cDNA (GenBank accession number: OK315661) was 535 bp in length, including a 5′-UTR of 36 bp, a 3′-UTR of 253 bp with a potential polyadenosine (ATTAAA), and an ORF of 246 bp encoding a 81 amino acid with a calculated molecular weight of ~9.03 kDa and a predicted isoelectric point of 10.11. The deduced amino acid sequence of Es-β-PDH containing a 25-residue signal peptide, a 33-residue precursor related peptide (PPRP), two proteolytic sites (KR, RR) at positions 59 and 80, and a 18-residue mature peptide (**Figure 1B**). Sequence alignments showed that the amidation site and two proteolytic sites are identical in different crustacean species, and the PDH mature peptides are conserved in crustaceans (**Figure S1B**). Phylogenetic analysis showed that Es-β-PDH is more closely related to crustacean β-PDHs than α-PDH, followed by pigment dispersing factor, which is also consistent with traditional classification (**Figure S2B**).

### Tissue Distribution of *Es-RPCH* and *Es-β-PDH* mRNA

**Figure 2:**
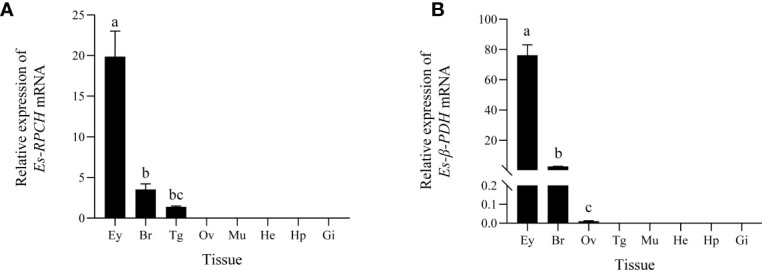
QRT-PCR detection of the tissue distribution of *Es-RPCH* **(A)** and *Es-β-PDH* **(B)** mRNAs in *E. sinensis*. Ey, eyestalks; Br, brain; Tg, thoracic ganglia; Ov, ovary; Mu, muscle; He, heart; Hp, hepatopancreas; Gi, gill. Different letters indicate significant differences (p <0.05)

QRT-PCR detection showed high expression of *Es-RPCH* and *Es-β-PDH* mRNA in the eyestalks, and moderate in brain. While weak expression of the *Es-RPCH* and *Es-β-PDH* was found in thoracic ganglia and ovary, respectively. No expression was found in other tested tissues (**Figure 2**).

### *ISH* Localization of *Es-RPCH* and *Es-β-PDH* mRNA in the Brain

**Figure 3:**
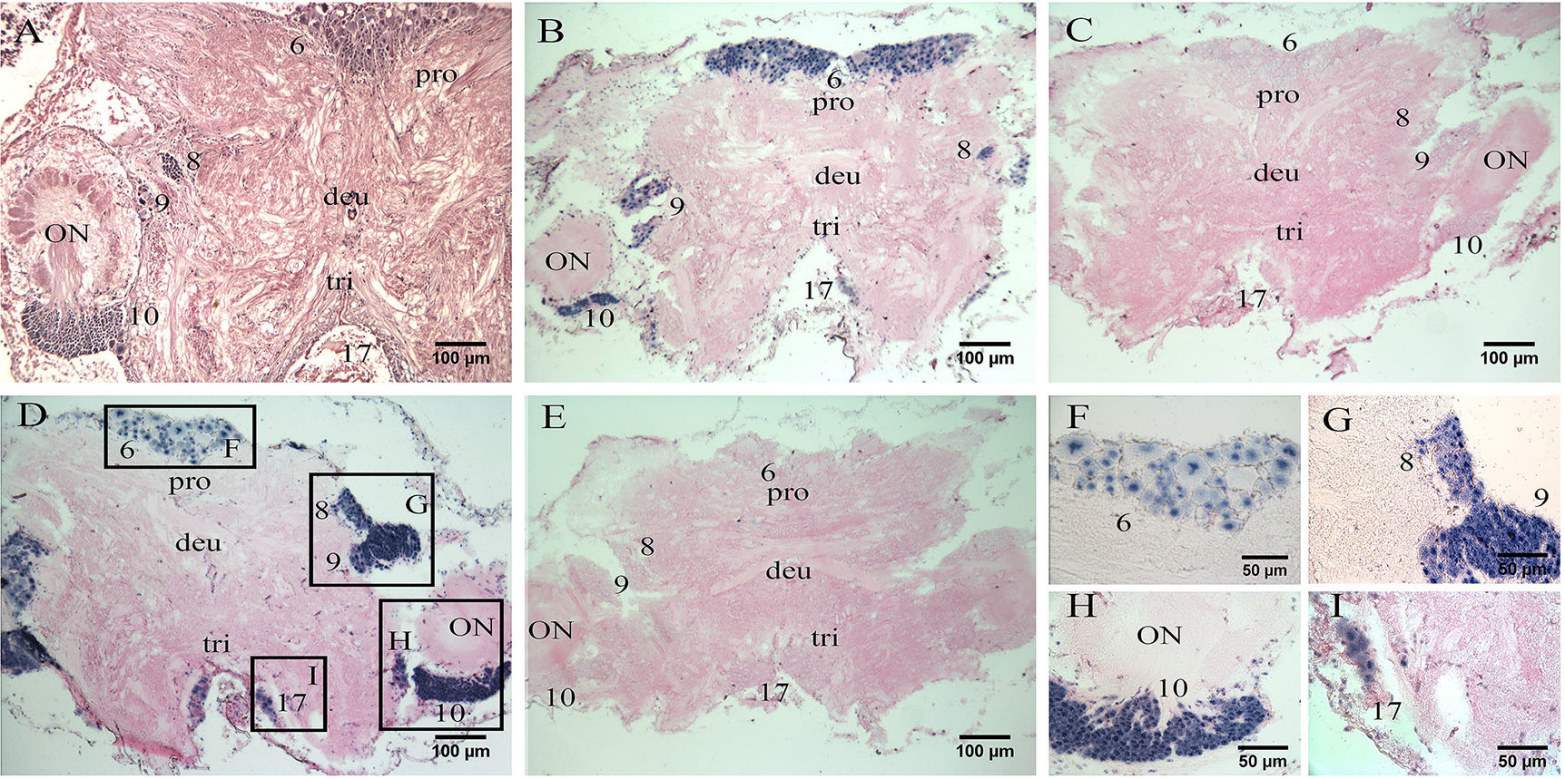
*In situ* hybridization (ISH) localization of *Es-RPCH* and *Es-β-PDH* mRNAs in the brain of *E. sinensis*. **(A)** Hematoxylin-eosin staining of a horizontal section of the brain showing the locations of neuronal clusters (numbers) and neuropils. **(B)** ISH using *Es-RPCH* antisense probes showing positive signals (blue) in various neuronal clusters of the brain. **(C)** Negative control of ISH using *Es-RPCH* sense probes. **(D)** ISH using *Es-β-PDH* antisense probes showing positive signals (blue) in various neuronal clusters of the brain. **(E)** Negative control of ISH using *Es-β-PDH* sense probes. Scale bars = 100 µm. **(F, I)** High magnification micrographs showing positive ISH signals in the cluster 6 **(F)**, clusters 8 and 9 **(G)**, cluster 10 **(H)**, and cluster 17 **(I)**. Scale bars= 50 µm. ON, olfactory neuropil; pro, protocerebrum; deu, deutocerebrum; tri, tritocerebrum.

Histological section showed that the mitten crab brain can be classified into protocerebrum, deutocerebrum, and tritocerebrum (36). Middle horizontal section of the brain in **Figure 3A** showed the locations of neuronal clusters (numbers) and neuropils. A strong positive signal of *Es-RPCH* was visualized in the cell clusters 6 in the protocerebrum, middle signal in cell clusters 8 in the protocerebrum, 9 and 10 in the deutocerebrum, but a weaker signal in cell clusters 17 in the tritocerebrum (36) (**Figures 3B, F**). In addition, a strong positive signal of *Es-β-PDH* was visualized in the cell clusters 8, 9, and 10, but weaker in cell clusters 6 and 17 (**Figures 3D, G–I**). No positive signal was detected in negative control sections with the sense-strand RNA probes (**Figures 3C, E**).

### Quantitative Expression of *Es-RPCH* and *Es-β-PDH* mRNA During Ovarian Development

**Figure 4:**
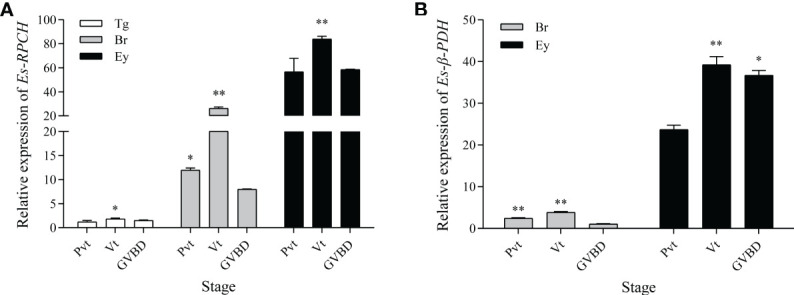
qPCR assay of the relative expression level of *Es-RPCH* **(A)** and *Es-β-PDH* **(B)** mRNA in the thoracic ganglia (Tg), brain (Br);, eyestalks (Ey) during ovarian development in *E. sinensis.* Ovarian stages are divided into previtellogenic (Pvt), vitellogenic (Vt) and germinal vesicle breakdown (GVBD) stages. The data are presented as the means + SD (*n = 3*). * represents significant difference (*P* < 0.05), and ** represents highly significant difference (*P* < 0.01).

As shown in **Figure 4**, qRT-PCR indicated that the abundance of *Es-RPCH* and *Es-β-PDH* mRNA were the highest in the eyestalks, and moderate in the brain at each ovarian developmental stage (**Figure 4A**). The relative expression level of *Es-RPCH* and *Es-β-PDH* mRNA were all dramatically increased in the eyestalks and brain and reach the highest at Vt stage, and then decrease at GVBD stage (*P <*0.01) (**Figure 4B**).

### Effect of Es-RPCH and Es-β-PDH Peptides on Ovarian Maturation *In Vivo*

**Figure 5:**
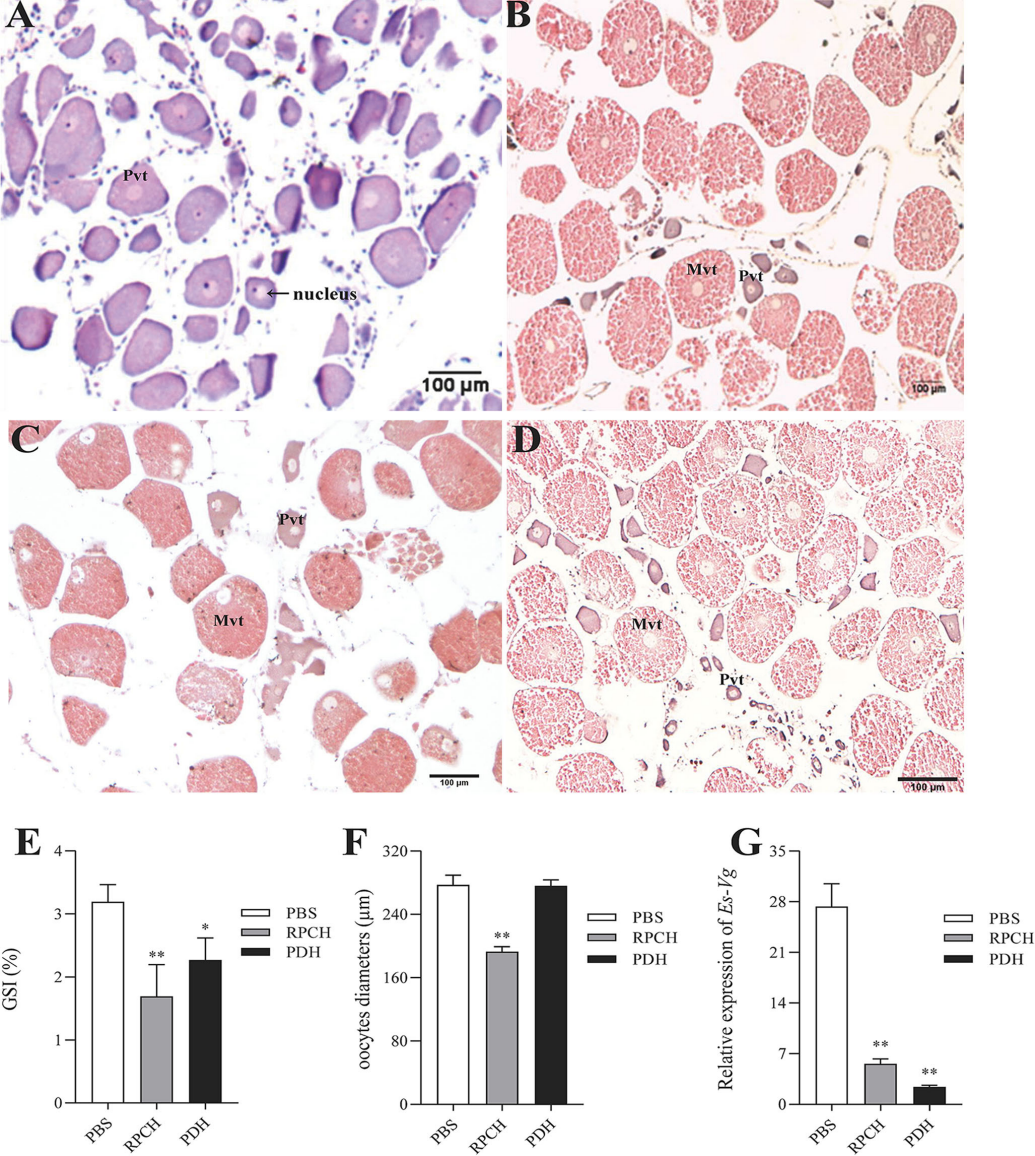
Effects of *in vivo* injection of Es-RPCH and Es-β*-*PDH peptides on the ovarian development in *E. sinensis*. Histological observation: Ovarian tissue sections at the first day **(A)** and the 30 st day **(B)** post injection of PBS; ovarian tissue sections at the 30 st day post injection of the Es-RPCH peptides **(C)** and Es-β*-*PDH peptides **(D)**. Inhibitory effects on the gonadosomatic index **(E)**, mean oocytes diameters **(F)**, and relative expression level of *Es-Vg* mRNA as revealed by qPCR **(G)**. Pvt, previtellogenesis. Mvt, middle vitellogenesis. The data are presented as means + SD (*n = 5*). Asterisks (*) and double asterisks (**) represent statistically significant difference as compared with PBS control (*P* < 0.05 and *P* < 0.01). Scale bar = 100 μm.

To investigate the exact role of the *Es-RPCH* and *Es-β-PDH* in the ovarian maturation in *E. sinensis*, the female individuals at Pvt stage were selected for injection of the synthesized Es-RPCH and Es-β*-*PDH peptides. At the beginning of experiment, the nucleus in the Pvt oocytes were transparent and were termed germinal vesicle (GV), while the ooplasm and nucleolus were notably stained in dark blue color by hematoxylin (**Figure 5A**). After administrated with injection of 30 days, the oocytes were visualized at Mvt stage in PBS, Es-RPCH, and Es-β*-*PDH groups. Due to the appearance of yolk, the ooplasm was stained in red by eosin (**Figures 5B–D**). Notably, in the Es-RPCH injection group, the GV visually migrated toward peripheral cytoplasmic membrane as compared with that of the Es-β*-*PDH and PBS groups (**Figures 5C, D**), in which GV situated in the center of oocyte. The GSI index and *Vg* expression in the ovary were all significantly decreased in the Es-RPCH and Es-β*-*PDH injection group when compared with the PBS control group (**Figures 5E, G**). The mean oocytes diameter was also smaller in the Es-RPCH group, but no significant differences between Es-β*-*PDH and the control groups (**Figure 5F**).

### Effect of Es-RPCH and Es-β*-*PDH Peptides on GVBD of Oocytes *In Vitro*

**Figure 6:**
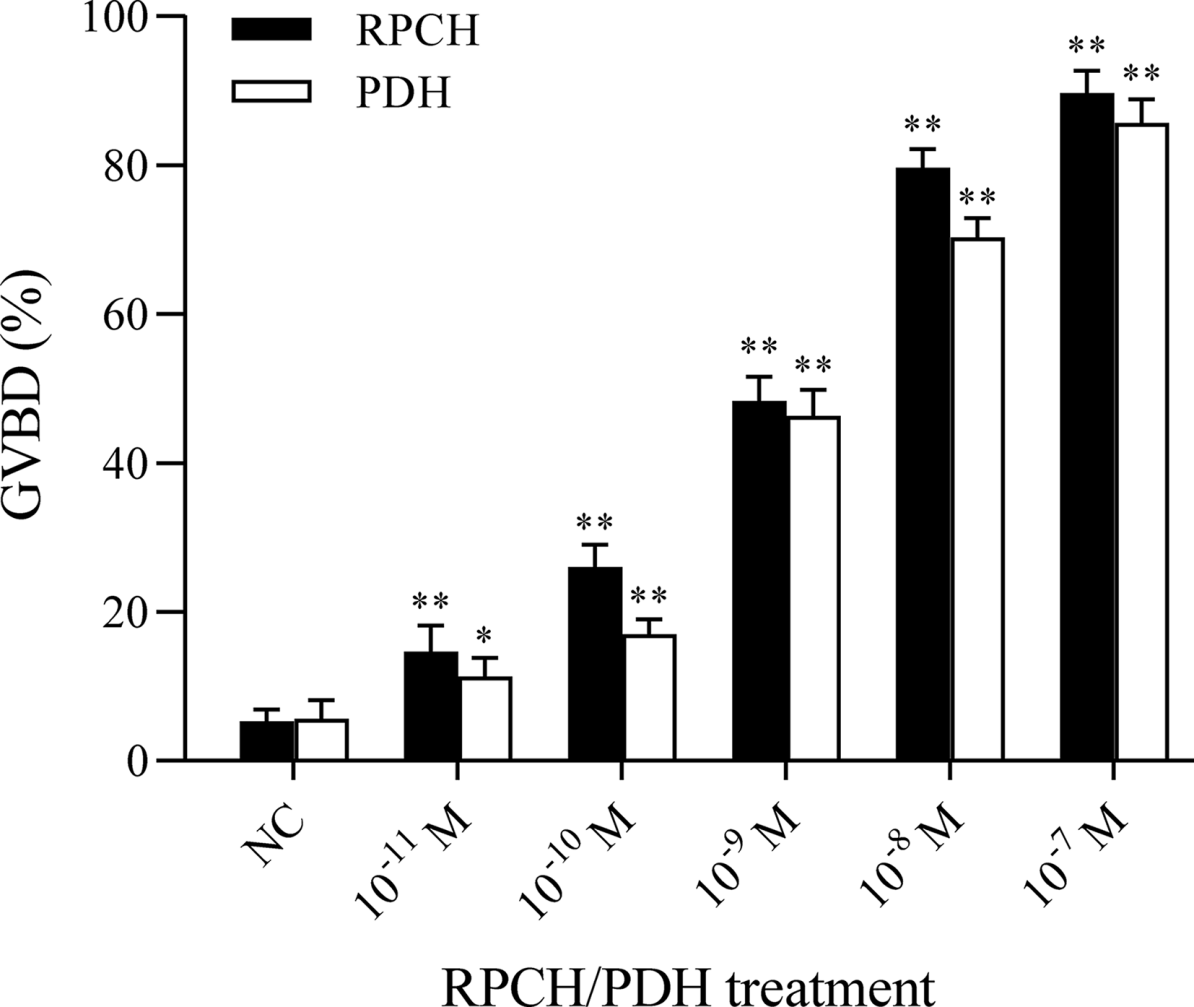
Effects of *in vitro* culture of the oocytes with the Es-RPCH and Es-β*-*PDH peptides on the germinal vesicle breakdown index in *E. sinensis*. A replicate from the control group (NC) was used as a calibrator. Bars represent mean + SD (*n = 5*). Asterisks (*) and double asterisks (**) represent values statistically different from the control group (*P* < 0.05 and *P* < 0.01).

To determine whether the *Es-RPCH* and *Es-β-PDH* can induce GVBD, oocytes at Lvt stage were selected to culture with the synthesized Es-RPCH and Es-β*-*PDH peptides. Results showed that the GVBD index in the Es-RPCH and Es-β*-*PDH group were all dramatically increased at the concentration of 10^-11^ M, 10^-10^ M, 10^-9^ M, 10^-8^ M, and 10^-7^ M as compared with control groups (*p* < 0.05). There was a significant dosage effect with the increasing concentration of Es-RPCH or Es-β*-*PDH. In addition, the GVBD index in Es-RPCH group was higher than in the Es-β*-*PDH in each concentration gradient (**Figure 6**).

### Ovarian Transcriptome Analysis

To probe the molecular basis of induction of GVBD by Es-RPCH and Es-β*-*PDH, nine ovarian cDNA libraries from the crabs administrated with PBS, Es-RPCH and Es-β*-*PDH were constructed and sequenced by Illumina HiSeq 4000 platform. More than 42 million raw reads were generated, and then about 33 million clean reads of every group were obtained after removing adapters as well as filtering the low-quality sequences. The Q20 percentage of each sample was over 97%, the Q30 percentage was over 92%, and the CG content was below 53.96% (**Table S2**), indicating that the sequencing data are high quality and credibility.

**Figure 7:**
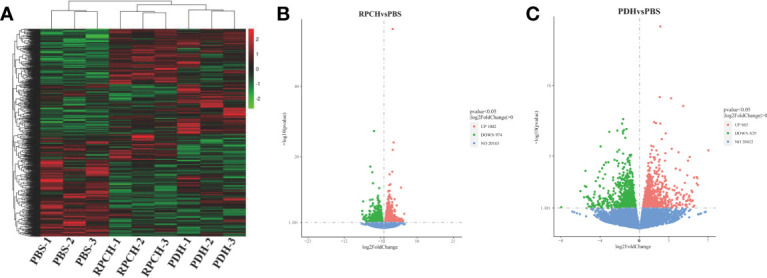
Comparative transcriptome analysis of the ovaries from the individuals (*n* = 3) injected with PBS, Es-RPCH and Es-β*-*PDH peptides. **(A)** A heat map of differentially expression genes (DEGs) in the ovaries administrated with PBS, Es-RPCH and Es-β*-*PDH peptides. The color scale indicates that the log10‐fold change from high (red) to low (green). **(B)** A volcano plot of DEGs between the Es-RPCH administrated and PBS control groups. **(C)** A volcano plot of DEGs between the Es-β*-*PDH administrate and PBS control groups. The upregulated, downregulated and non-regulated genes were indicated by red, green and blue dots, respectively.

**Figure 8:**
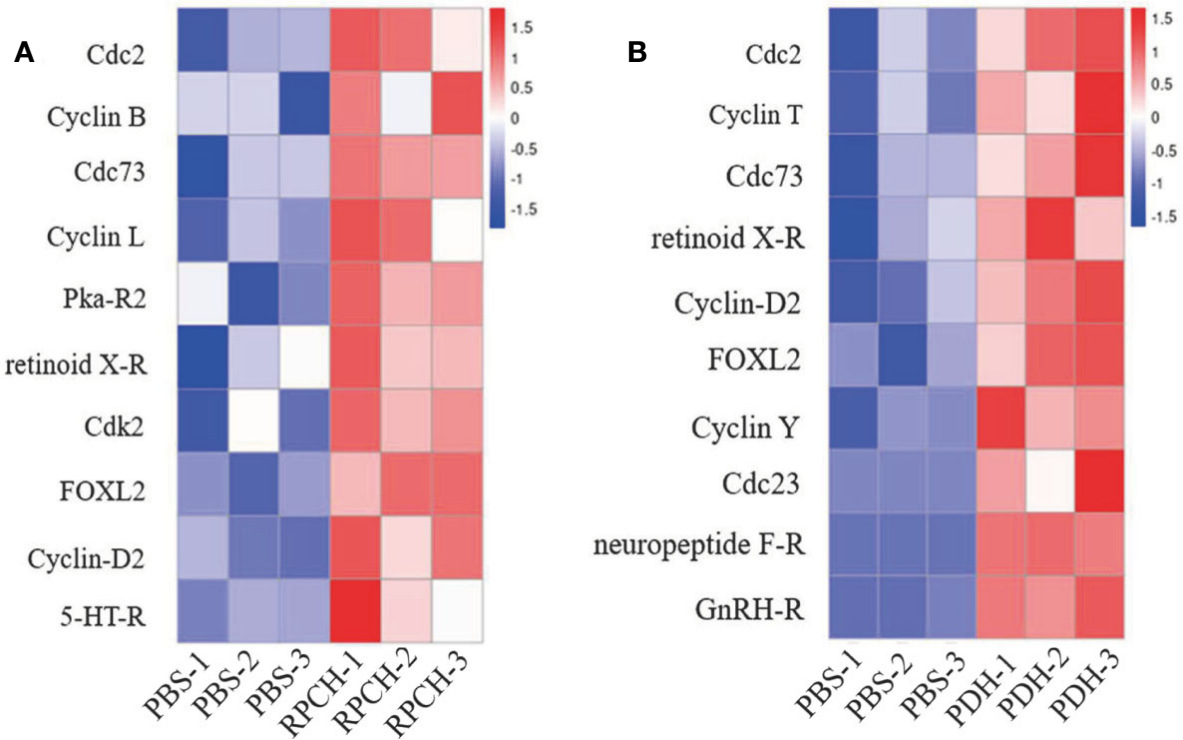
Heat maps of the DEGs related to oocyte meiosis and maturation. **(A)** The DEGs relative expression levels between the Es-RPCH administrated and PBS control groups (*n = 3*). **(B)** The DEGs relative expression levels between the Es-β*-*PDH administrated and PBS control groups (*n = 3*). The color reflects relative expression levels from high (red) to low (green).

In comparison with PBS group, a total of 1976 differentially expressed genes (DEGs) including 1002 up- and 974 down-regulated genes were identified in the Es-RPCH group, as well as1714 DEGs including 885 up- and 829 down-regulated genes in the Es-β*-*PDH group (**Figure 7**). GO analysis indicated DEGs from the Es-RPCH and Es-β*-*PDH groups were all classified into three ontologies: molecular function, biological process, and cellular component. Respectively, DEGs in the Es-RPCH group were mainly assigned into DNA binding, DNA integration and biosynthetic processes (**Figure S3A**), while in the Es-β*-*PDH group DEGs were mainly assigned into DNA integration, small molecule metabolism process, and monosaccharide metabolism process (**Figure S3C**). In addition, KEGG pathway analysis showed that DEGs in the Es-RPCH group were statistically enriched some pathways, including TGF‐β signaling pathway, oocyte meiosis, and Wnt signaling pathway, and so on. (**Figure S3B**), while in the Es-β*-*PDH group the DEGs were mainly enriched into TGF‐β signaling pathway, pyruvate metabolism and insect hormone biosynthesis, and so on (**Figure S3D**). Interestingly, some DEGs related to meiosis such as cell division cycle 2 kinase (Cdc2) and cyclin proteins as well as 5-HT-R were identified to be highly expressed in the Es-RPCH and Es-β*-*PDH groups as compared with the PBS control (**Figure 8A**). Additionally, the DEGs related to gonadal maturation such as foxl2, retinoid-X receptor (RXR), neuropeptide F-R, and GnRHR were also detected to be highly expressed in the Es-RPCH and Es-β*-*PDH groups (**Figure 8B**).

## Discussion

In the present study, the full-length cDNA of *Es-RPCH* and *Es-β-PDH* were identified and characterized in the brain of *E. sinensis*. Phylogenetic analysis indicated that the Es-RPCH belongs to the adipokinetic hormone (AKH)/RPCH family proteins and grouped into GnRHs superfamily (**Figure S2A**). This result was also supported by several structural characteristics of RPCH. Firstly, the signal peptide was demonstrated critical for guiding the AKH precursor into the secretory pathway, cleaved post-translationally, and then convert the prohormone into a prohormone, which was predicted in Es-RPCH (37). Secondly, the glycine residue (G) preceding the dibasic cleavage site (KR) as an amine donor yielding an amidated tryptophan (W-NH2) to be amidation at the C-terminus ends in AKHs, which is important for increasing binding affinity to the receptor and were also predicted in Es-RPCH (13, 38, 39). Insect AKHs was proposed as the cognate ligand of a GnRH receptor in *C. elegans* involving in egg laying behavior (40). Owing to the co-evolution of members of the GnRH superfamily and their receptors, their diverse functions seem to overlap including reproduction (41). Thirdly, the Es-RPCH mature peptide shares the 100% identity with those of other crustacean RPCHs and shares the 75% identity with insect AKHs (**Figure S1A**), indicating that the structure and function of RPCHs could be conserved during evolution. In contrast, the Es-β-PDH has the conserved PDH mature peptides sequence with identical amidation and proteolytic sites (**Figure S1B**). Phylogenetic analysis showed that Es-β-PDH is clustered with insects pigment dispersing factor (PDF)-commonly considered an equivalent of crustaceans PDH (**Figure S2A**), which has implication in regulating reproduction and ovarian maturation (42).

Many pioneer studies suggested that RPCH and PDH are generated in the eyestalks (18, 19, 28). Our present study revealed the *Es-RPCH* and *Es-β-PDH* were also produced in the brain besides eyestalks (**Figure 2**). The hybridization signal of *Es-RPCH* were widely distributed in the neuronal clusters 6, 8, 9, 10, and 17 of the brain (**Figure 3**), which is identical with the expression pattern of *RPCH* in the brain of *Scylla olivacea* (18). The ubiquitously distribution in neuronal clusters of the brain indicated that *Es-RPCH* may perform various important physiological functions in crustaceans, which refer to the neuronal clusters 9 and 10 of brain playing an important role in visual and chemical sensations reception, as well as feeding and reproductive behaviors regulation in crustaceans (43, 44). Interestingly, similarly results were also found in *Es-β-PDH* indicated that their biological function maybe overlapping.

To explore whether *Es-RPCH* and *Es-β-PDH* are involved in the ovarian maturation in *E. sinensis*, we firstly examined the expression profiles of *Es-RPCH* and *Es-β-PDH* in neural tissues during ovarian development. QPCR results shows both *Es-RPCH* and *Es-β-PDH* are significantly increased and highest in vitellogenic stage, decreased in germinal vesicle breakdown stage (**Figure 4**), suggesting the potential role of *Es-RPCH* and *Es-β-PDH* in ovarian maturation. Then the female individuals at Pvt stage were selected for injection of synthetic Es-RPCH and Es-β-PDH peptides. The results showed *Es-RPCH* can induce the germinal vesicles shifting from center to peripheral plasma membrane in vitellogenic oocyte (**Figure 5**). *In vitro* culture experiments further revealed that Es-RPCH peptides can induce germinal vesicle breakdown in the oocytes at Lvt stage (**Figure 6**). The mean oocyte diameter, *EsVg* expression and GSI, however, were all significantly decreased after Es-RPCH peptides injection (**Figure 5**), indicating that the *Es-RPCH* inhibit the vitellogenesis and induce oocyte meiotic maturation. At the end of vitellogensis, the oogenesis enters into the final meiotic maturation stage. The vitellogenesis is terminated and the germinal vesicles begin to migrate toward peripheral plasma membrane and ultimately break down. Thus, we deduced that *Es-RPCH* could function at the transition from vitellogenesis to final meiotic maturation of oocytes. Like *Es-RPCH*, the *Vg* expression and GSI were also notably decreased when injection of Es-β-PDH peptide *in vivo*. Although no shifting of germinal vesicle was observed, the Es-β-PDH peptide can also induce germinal vesicle breakdown of oocyte at Lvt stage when culture *in vitro* (**Figure 6**). It is worth to note that our results are inconsistent with the previous studies in *S. paramamosain* (24), *P. clarkii* (22), and *L. vannamei* (19), in which the injection of RPCH can increase mean oocyte diameter, *Vg* expression and GSI. As an eyestalk hormone, to our knowledge, the inductive effect of RPCH in vitellogenesis is unexplained and the inhibitory effect is more reasonable since it is well known that removal of eyestalks can induce the vitellogensis.

To better understand the molecular regulatory basis of the *Es-RPCH* and *Es-β-PDH* in ovarian maturation, we next performed ovarian transcriptome analysis. KEGG pathway analysis showed that reproduction-related pathways such as the Wnt and TGF‐β signaling pathway were enriched (45, 46) (**Figure S3**). Interestingly, some key genes involved in meiosis and ovarian development, such as cyclin B, Cdc2, 5-HT-R, RXR and FOXL2, were detected to be upregulated in Es-RPCH group (**Figure 8**). Cdc2 kinase and cyclin B are components of M-phase promoting factor (MPF), a heterodimer responsible for the final meiotic maturation of oocyte (30, 47–49). Furthermore, 5-HT binding to 5HT-R can induce GVBD in mud crab *S. paramamosain* (50). RXR and FOXL2 can regulate *Vg* expression and ovarian development in *S. paramamosain* and *P. trituberculatus* (51–54). Similar results were also found in the comparative transcriptome analysis of Es‐β-PDH injection group, suggesting that *Es-RPCH* and *Es-β-PDH* might have similar regulatory pathway for inducing oocyte meiotic maturation and ovarian maturation in *E. sinensis*.

## Conclusion

The full-length cDNA of *Es-RPCH* and *Es-β-PDH* were identified and functionally characterized in *E. sinensis*. The synthestic Es-RPCH and Es-β-PDH peptides can induce the germinal vesicles breakdown *in vitro*, but downregulate the *Vg* expression and decreased the GSI *in vivo*. In addition, comparative ovarian transcriptome analysis further revealed that meiosis-related genes were significantly upregulated in response to Es-RPCH and Es-β-PDH peptides. Our study provided the first evidence of the stimulating effect of *Es-RPCH* and *Es-β-PDH* in the oocyte meiotic maturation in *E. sinensis.*

## Data Availability Statement

The datasets presented in this study can be found in online repositories. The names of the repository/repositories and accession number(s) can be found in NCBI. RPCH group; SRR16603372 PDH group; SRR16603373 control group; SRR16603374.

## Ethics Statement

Ethical review and approval was not required for the animal study because the mitten crab *E. sinensis* is not an endangered or protected species, and permission to perform experiments involving this species is not required in China. Written informed consent for participation was not obtained from the owners because the mitten crab *E. sinensis* is not an endangered or protected species, and permission to perform experiments involving this species is not required in China.

## Author Contributions

L-LW performed the experiments and analyzed data. T-TC wrote the manuscript and analyzed data. B-YL instructed oocytes culture experiments. G-FQ designed this study, analyzed data and revised the manuscript. All authors contributed to the article and approved the submitted version.

## Funding

This research was supported by the National Key R&D Program of China (project number 2018YFD0900201) and the Natural Science Foundation of China (project number 41976103, 41476130).

## Conflict of Interest

The authors declare that the research was conducted in the absence of any commercial or financial relationships that could be construed as a potential conflict of interest.

## Publisher’s Note

All claims expressed in this article are solely those of the authors and do not necessarily represent those of their affiliated organizations, or those of the publisher, the editors and the reviewers. Any product that may be evaluated in this article, or claim that may be made by its manufacturer, is not guaranteed or endorsed by the publisher.
