# Supplementary material for: Evidences for Red Pigment Concentrating Hormone (RPCH) and Beta-Pigment Dispersing Hormone (β-PDH) Inducing Oocyte Meiotic Maturation in the Chinese Mitten Crab, Eriocheir sinensis
Source: Front Endocrinol (Lausanne). 2021 Dec 16;12:802768. doi: 10.3389/fendo.2021.802768 (PMC8716682; doi:10.3389/fendo.2021.802768)
Supplement: Supplementary file 1 [file DataSheet_1.docx]

**Supplementary material**

**Fig. S1** Multiple alignments of the deduced amino acid sequences of Es-RPCH (A) and Es-β*-*PDH (B) with other known species. The mature peptides are indicated by asterisk (*).The amidation site and postulated dibasic cleavage site (KR) of Es-RPCH and two predicted proteolytic sites (KR and RR) of Es-β-PDH are indicated by white and black triangles and boxes, respectively. Species abbreviations in the sequence labels are as follows: (A): Es, *Eriocheir sinensis*; So, *Scylla olivacea*; Sp, *Scylla paramamosain*; Pv, *Penaeus vannamei*; Cp, *Cherax quadricarinatus*; Mr, *Macrobrachium rosenbergii*; Nn, *Nephrops norvegicus*; Lc, *Lucilia cuprina*; Tc, *Tribolium castaneum*; Ls, *Lucilia sericata*. (B): Es, *Eriocheir sinensis*; Sp, *Scylla paramamosain*; Nn, *Nephrops norvegicus*; Cp, *Cancer productus*; Ha, *Homarus americanus*; Cq, *Cherax quadricarinatus*; Pj, *Penaeus japonicus*. GenBank accession numbers for each sequence are listed in **Figure S2**.

**Fig. S2** Phylogenetic analysis of RPCH (A) and PDH (B) from different species using the Neighbour-joining method (1000 bootstrap replicates). The amino acid sequences used for the phylogenetic analysis were downloaded from the GenBank database with the accession numbers in the brackets. The Es-RPCH and Es-β*-*PDH were in bold.

**Fig. S3** GO and KEGG enrichment analysis of the gene differential expression (DEGs) in *E. sinensis*. GO annotation in biological processes, molecular functions, and cellular components showing the distribution of DEGs in Es-RPCH (A) and Es-β*-*PDH (C) administrated groups. Classification chart of KEGG analysis of DEGs in Es-RPCH (B) and Es-β*-*PDH (D) administrated groups. The bubble color represents the significance of enrichment degree and the bubble size represents the number of genes.

**

**

**Fig. S1**


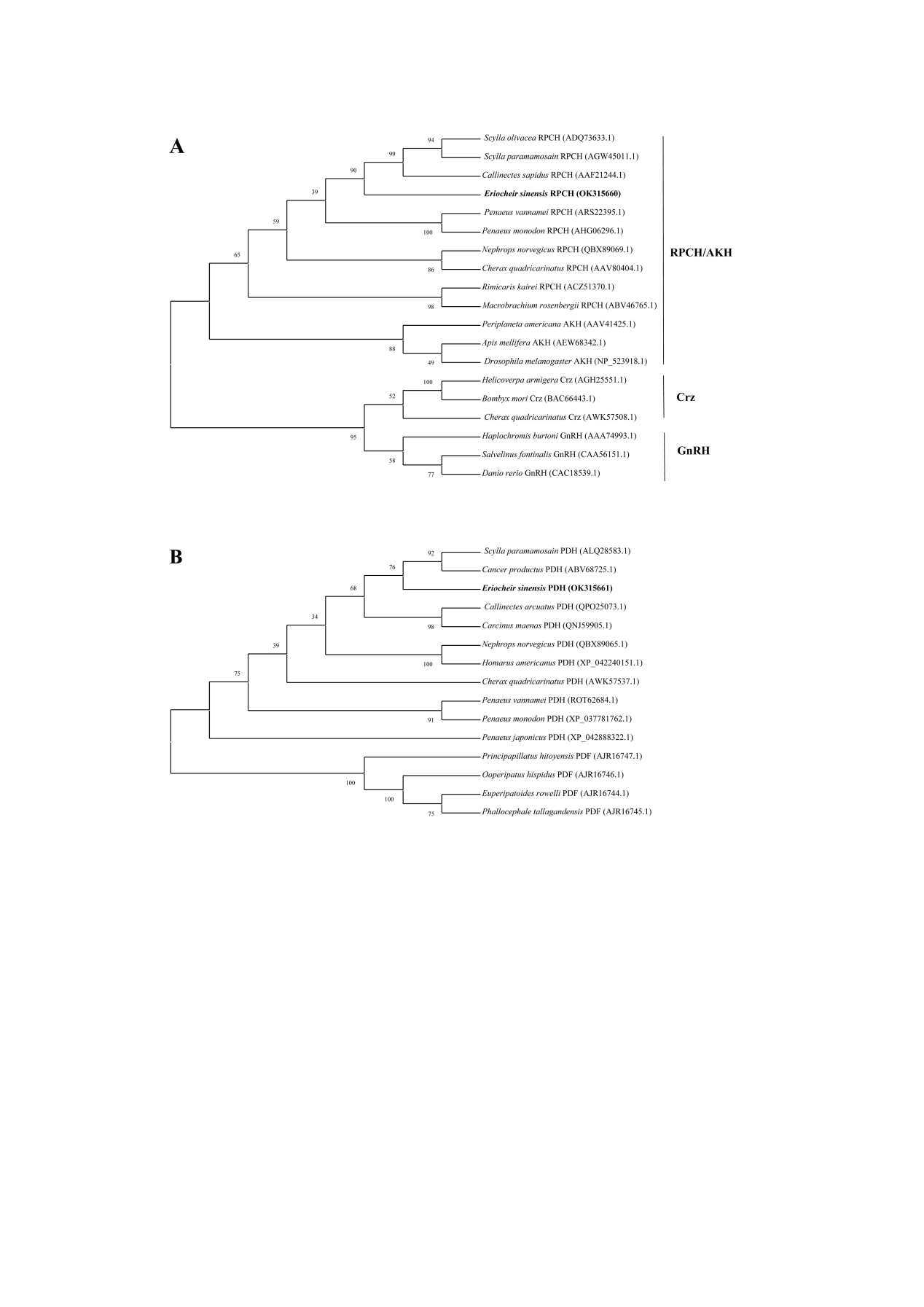


**Fig. S2**


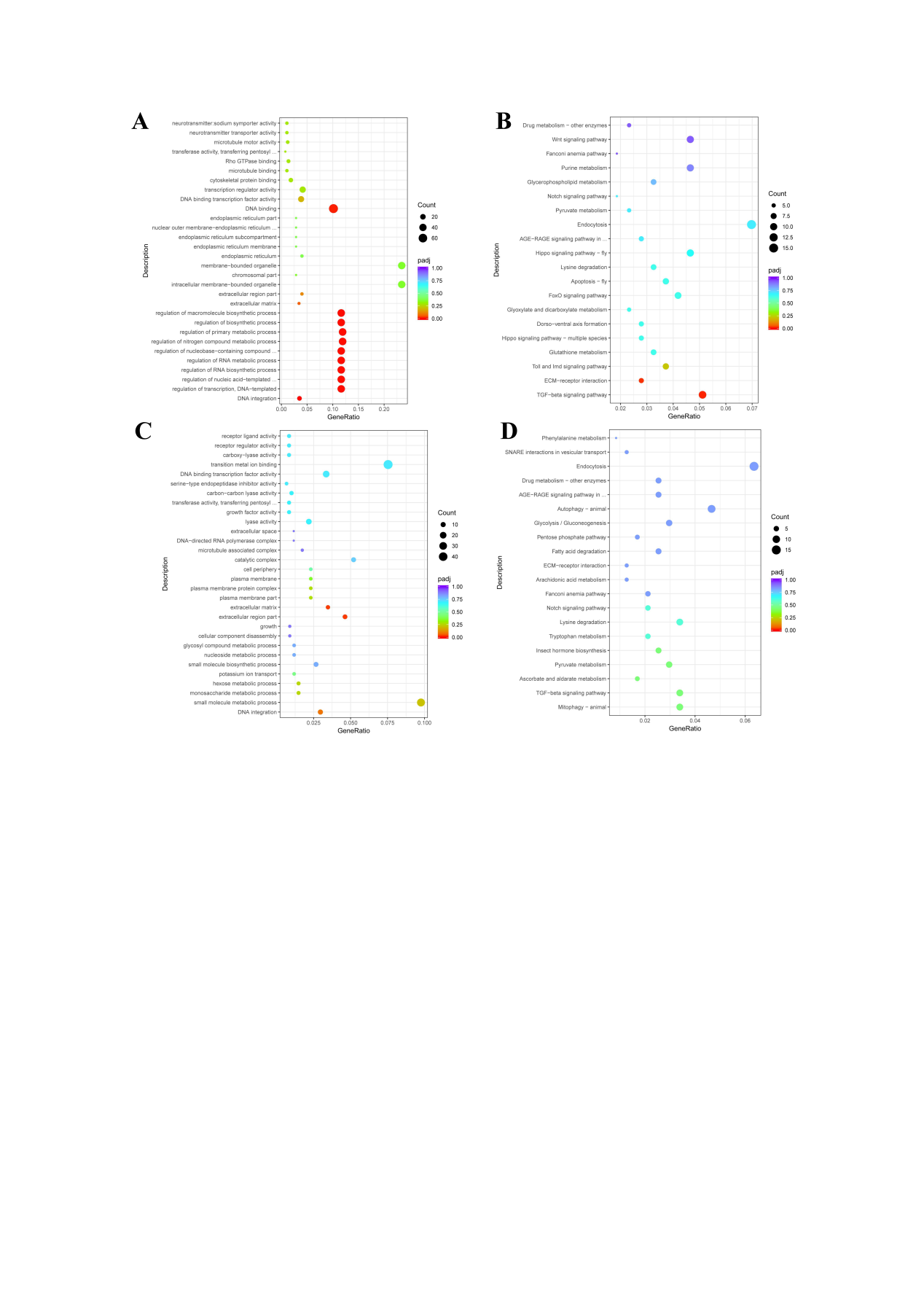


**Fig. S3**

**Table S1. PCR primers used in this study**

| Primer | Sequence (5'→3') | Purpose |
| --- | --- | --- |
| Es-RPCH-F | CGCAACACAACACAGCCCAC | RT-PCR |
| Es-RPCH-R | GACTCCTATCCAAATCATCCAGCC | RT-PCR |
| Es-PDH-F | CCCTACGACACCCCAACACA | RT-PCR |
| Es-PDH-R | TGAAGAAGCTCTCAACGCG | RT-PCR |
| Es-RPCH-3F | CAACACAGCCCACTCGACG | 3' RACE |
| Es-PDH-3F | TCACCCCTACGACACCCCAA | 3' RACE |
| Es-RPCH-5R | GAGTCGGTAGATGTGCATGACGG | 5' RACE |
| Es-PDH-5R | ATCTGCCGTGCCAGTTCAGC | 5' RACE |
| UPM | CTAATACGACTCACTATAGGGCAAGCAGTGGTATCAACGCAGAGT | 3'/5' RACE |
| UPS | CTAATACGACTCACTATAGGGC | 3'/5' RACE |
| qRPCH-F | CACATCTACCGACTCATCAAG | qPCR and cloning for ISH probes |
| qRPCH-R | GCAAAGACTCCTATCCAAAT | qPCR and cloning for ISH probes |
| qPDH-F | CCCCCTATCCACATACTTCTACA | qPCR and cloning for ISH probes |
| qPDH-R | ACATGAGAGCCTGACCTTCG | qPCR and cloning for ISH probes |
| β-actin-F | CCTCACCCTCAAATACCCCAT | qPCR |
| β-actin-R | GGGGTGTTGAAGGTCTCGGA | qPCR |
| qVg-F | AAGTGAACAACCGTGTGGCA | qPCR |
| qVg-R | CAGCAAATGGCTCAGGATAA | qPCR |

**Table S2. Evaluation of data quality of transcriptome of each sample**

| Sample | Raw reads | Clean reads | Q20（%） | Q 30（%） | GC (%) |
| --- | --- | --- | --- | --- | --- |
| PBS-1 | 45597834 | 44111706 | 97.25 | 93.18 | 52.92 |
| PBS-2 | 43398762 | 42062538 | 97.23 | 93.17 | 53.96 |
| PBS-3 | 48897470 | 47387812 | 97.14 | 92.96 | 53.19 |
| Es-RPCH-1 | 47409254 | 45867470 | 97.16 | 92.98 | 53.50 |
| Es-RPCH-2 | 42492834 | 41253696 | 97.39 | 93.46 | 53.71 |
| Es-RPCH-3 | 45730476 | 44178874 | 97.40 | 93.44 | 53.28 |
| Es-PDH-1 | 48162028 | 46886274 | 97.35 | 93.42 | 53.65 |
| Es-PDH-2 | 45992362 | 44621792 | 97.34 | 93.33 | 53.25 |
| Es-PDH-3 | 46288004 | 45007448 | 97.23 | 93.04 | 52.90 |
